# The gap height in open wedge high tibial osteotomy is not affected by the starting point of the osteotomy

**DOI:** 10.1186/s12891-023-06478-8

**Published:** 2023-05-11

**Authors:** Moritz Mederake, Georgios Eleftherakis, Daniel Schüll, Fabian Springer, Nicola Maffulli, Filippo Migliorini, Christian Konrads

**Affiliations:** 1 Department of Orthopaedic Surgery, University Hospital Tübingen, Tübingen, Germany; 2 Department of Trauma and Reconstructive Surgery, BG Klinik, University of Tübingen, Tübingen, Germany; 3 Department of Radiology, University of Tübingen, Tübingen, Germany; 4 Department of Medicine, Surgery and Dentistry, University of Salerno, Baronissi, SA 84081 Italy; 5 School of Pharmacy and Bioengineering, Keele University Faculty of Medicine, Stoke on Trent, ST4 7QB England; 6 Barts and the London School of Medicine and Dentistry, Centre for Sports and Exercise Medicine, Queen Mary University of London, Mile End Hospital, London, E1 4DG England; 7 Department of Orthopaedic, Trauma, and Reconstructive Surgery, RWTH University Hospital, Pauwelsstraße 30, 52074 Aachen, Germany; 8 Department of Orthopaedic and Trauma Surgery, Academic Hospital of Bozen, Bozen, 39100 Italy; 9 Department of Orthopaedics and Traumatology, Helios Hanseatic Hospital Stralsund, Stralsund, Germany

**Keywords:** Frontal malalignment, Frontal realignment, Correction osteotomy, Varus, Valgus, Osteotomy planning

## Abstract

**Introduction:**

In open-wedge high-tibial-osteotomy (OWHTO), most surgeons use a preoperative planning software and realise that they should match the intraoperative alignment correction with the preoperative plan. We aimed to determine whether there is a difference in osteotomy gap height when starting the OWHTO either 3 or 4 cm distal to the joint line. This should help to clarify whether the osteotomy starting point must exactly match the preoperative planning.

**Methods:**

25 patients with constitutional varus alignment were planned for OWHTO. Long-leg-standing-radiographs and mediCAD-software were used. Osteotomy was planned to a neutral Hip-Knee-Ankle angle (HKA) of 0°. The osteotomy-starting-point was either 3 or 4 cm distal to the medial joint line. The following angles were compared: mechanical medial proximal tibial angle (mMPTA), mechanical lateral distal femoral angle (mLDFA), joint line conversion angle (JCA), mechanical Tibio-Femoral angle (mTFA) or Hip Knee Ankle (HKA) angle.

**Results:**

25 Patients (18 males, 7 females) had a mean age of 62 ± 16.6 years and showed a varus-aligned leg-axis. The HKA was − 5.96 ± 3.02° with a mMPTA of 82.22 ± 1.14°. After osteotomy-planning to a HKA of 0°, the mMPTA was 88.94 ± 3.01°. With a mean wedge height of 8.08 mm when locating the osteotomy 3 cm and a mean wedge height of 8.05 mm when locating the osteotomy 4 cm distal to the joint-line, there was no statistically significant difference (p = 0.7).

**Conclusion:**

When performing an OWHTO aiming towards the tip of the fibula, the osteotomy starting point does not need to exactly match the planned starting-location of the osteotomy. A starting-point 1 cm more distal or proximal than previously determined through the digital planning does not alter the size of the osteotomy gap needed to produce the desired amount of correction.

## Introduction

Open wedge high tibial osteotomy (OWHTO) for correction of varus deformity of the knee is a well-established method to redistribute loads in the axial plane [1–4]. Proper patient selection, deformity analysis, and preoperative planning are crucial for a successful procedure [2, 5, 6]. In the past two decades, advances in digitalization has led to computer based preoperative deformity analysis and osteotomy planning using imaging, improving the accuracy of malalignment correction [7].

In OWHTO, the starting point for the oblique ascending osteotomy is in a range of 3 to 6 cm distal to the medial knee joint line and is limited by the surrounding anatomical structures [8, 9]. In digital planning programs, this can be planned and visualized preoperatively, and the basis of the wedge (length of the osteotomy gap height) can be measured. To match the intraoperative alignment correction with the preoperative plan, the measurement of the gap height is commonly used [10]. However, little is known about the effects of the osteotomy starting point at a different location intraoperatively than what was planned preoperatively. There is a lack of knowledge about whether gap measurement techniques can be applied regardless of the medial starting point location.

This study investigated the influence of the osteotomy starting point on wedge basis length and wedge angle. The outcome of interest was to investigate whether the osteotomy position at 3 or 4 cm distal to the medial knee joint line exerts an influence on the surgical outcome. It was hypothesized that a difference in wedge basis length and wedge angle for both positions exists.

## Methods

### Study cohort

The present study was conducted at the Department of Orthopedic Surgery of the University Hospital of Tübingen, Germany. Patients were prospectively recruited between January 2021 and January 2022. The digital planning of 25 patients planned for OWHTO for valgisation of a varus malalignment was evaluated. Inclusion criteria were medial compartment knee osteoarthritis and varus deformity with a mechanical medial proximal tibia angle (mMPTA) of < 85°. Exclusion criteria were prior limb realignment surgeries or previous arthroplasty of the lower extremities. All patients signed a written informed consent to participate in the present study. All procedures were performed in accordance with the Declaration of Helsinki and were approved by the Ethics Committee of the University of Tübingen, Germany (ID: 110/2021BO2).

### Imaging

Plain radiographs were obtained according to the method of Paley et al [10]. Full weight bearing long leg radiographs were performed with a steel reference ball with 25 mm diameter positioned closed to the knee for calibration. The X-ray beam was centered at the knee joint line at a distance of 305 cm to a long cassette. Each radiograph was exported from the Picture archiving and communication systems (PACS) image storage, transferred in DICOM (Digital Imaging and Communications in Medicine), and imported to mediCAD (Hectec GmbH, Landshut, Germany).

### Measuring and planning

Long leg standing radiographs were measured, and the OWHTO was planned by an experienced orthopedic surgeon using mediCAD software. With the 25 mm steel reference ball, the radiographs were calibrated utilizing the three-point method. The next step was to define the centre of the hip. Further important landmarks to be marked are the apex of the greater trochanter, the condyles and epicondyles of the femur and tibia, and the joint line and the centre of the talus. Additionally, the anatomical axes of the femur and tibia are defined. With these parameters, the software calculated the following angles:

- Mechanical medial proximal tibial angle (mMPTA).
- Mechanical lateral distal femoral angle (mLDFA).
- Joint line conversion angle (JCA).
- Mechanical Tibio-Femoral angle (mTFA) or Hip Knee Ankle (HKA) angle.

**Fig. 1:**
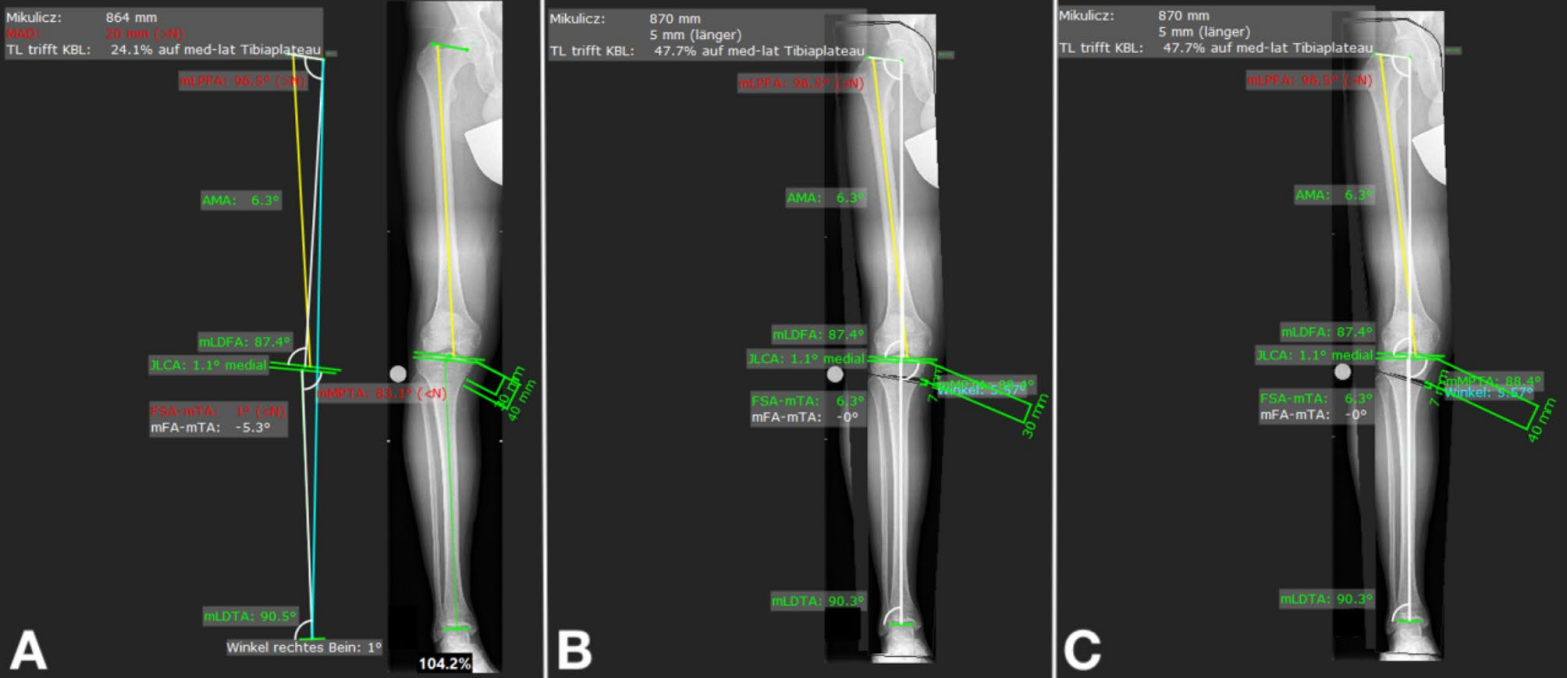
Planning of an open wedge high tibial osteotomy in the right knee on a full-length standing radiograph. (**A**) Before osteotomy, the frontal alignment is 5.3° of varus with an mMPTA of 83.1°. Osteotomy (**B**) 3 cm and (**C**) 4 cm distal to the medial joint line is planned to produce a neutral frontal alignment of 0°. The osteotomy gap is measured for both starting points (mMPTA: mechanical Medial Proximal Tibia Angle)

After deformity analysis, the OWHTO was planned: to measure the wedge basis length (osteotomy gap height), firstly the location of the osteotomy and the hinge point had to be defined. This was performed twice to compare two different positions of the osteotomy; (1) with the starting point of the osteotomy 3 cm distal, and (2) with the starting location 4 cm distal to the medial knee joint line at the proximal tibia (Fig. 1). The osteotomy line aims at the tip of the fibular head. The angulation correction axis (ACA) lies in the osteotomy plane. The wedge angle was then calculated by the software to correct the malalignment to a neutral alignment with an HKA of 0°. The correction angle and the length of the wedge basis were recorded for both planned osteotomies, and compared with each other (Fig. 1).

### Statistical analysis

Statistical analyses were conducted using IBM SPSS Version 20 and Microsoft Office Excel (Microsoft Corp., Redmond, WA, USA). Distributions of variables within the groups were assessed by histograms and a Shapiro-Wilk test and a parametric approach was chosen. Continuous variables are presented as medians and ranges, and categorical variables as frequencies. Comparison between groups was performed by paired t-test or Wilcoxon-test as appropriate. All reported p-values are two-sided with a significance level of 0.05, and they have not been adjusted for multiple testing.

## Results

25 patients were included in the present investigation. There were 18 men and 7 women with a mean age of 62 ± 16.6 years. The measured angles of the lower extremity before and after osteotomy planning are shown in Table 1.

**Table 1:** Relevant radiographic values were measured using long-leg standing radiographs and mediCAD software before and after open wedge High Tibial Osteotomy planning (HKA, Hip-Knee-Ankle angle; mMPTA, mechanical Medial Proximal Tibia Angle; mLDFA, mechanical Lateral Distal Femur Angle; JLCA, Joint Line Conversion Angle; OWHTO, open wedge High Tibial Osteotomy

Endpoints	Before osteotomy	3 cm below joint line	4 cm below joint line
HKA [°]	-5.96 ± 3.02	0.0 ± 0.0	0.0 ± 0.0
mMPTA [°]	82.22 ± 1.14	88.94 ± 3.01	88.94 ± 3.01
mLDFA [°]	86.98 ± 2.19	86.98 ± 2.19	86.98 ± 2.19
JLCA [°]	2.51 ± 1.60	2.51 ± 1.60	2.51 ± 1.60
Osteotomy gap height [mm]	-	8.08 ±	8.05 ±

**Fig. 2:**
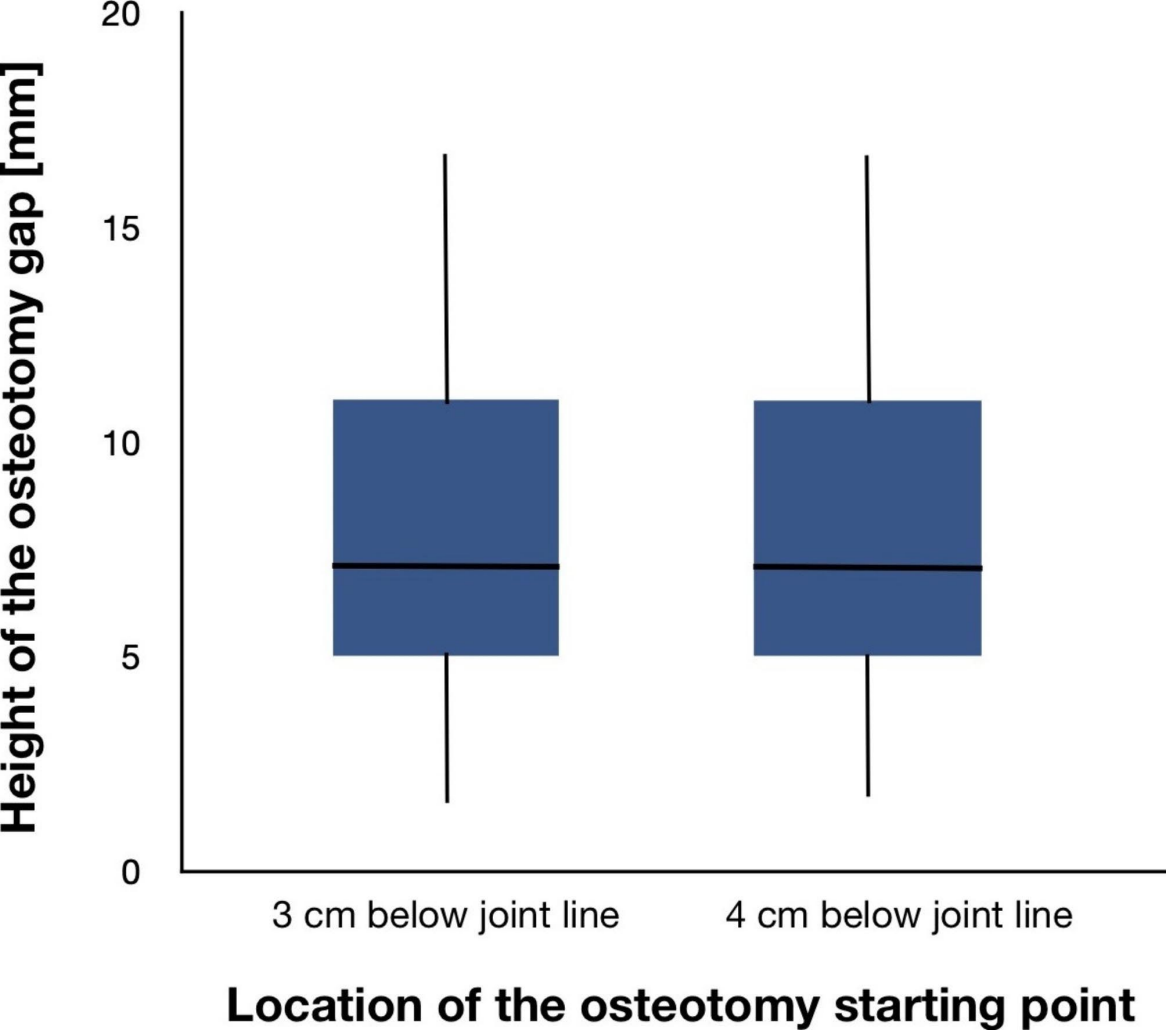
Comparison of osteotomy gap height (wedge basis height) when planning the OWHTO either 3 or 4 cm distal to the medial knee joint line

With a mean wedge height of 8.08 mm when locating the osteotomy 3 cm and a mean wedge height of 8.05 mm when locating the osteotomy 4 cm distal to the joint line, there was no statistically significant difference (p = 0.7) (Fig. 2). The mean absolute difference between both measurements was 0.0228 mm with a range from 0.01 to 0.11 mm. There was also no difference in mean wedge angle with 6.14° for both locations of the osteotomy (p = 0.7).

## Discussion

Valgisation osteotomies around the knee, including OWHTO, produce a major impact on medial compartment osteoarthritis of the knee and help to preserve the joint [11–14]. There are three essential keys important for the success of this surgery: correct indication, precise preoperative planning, and accurate surgery. Preoperative planning and the implementation of the planning during surgery need to go hand in hand, especially when using gap measurement techniques to control the alignment correction. Therefore, we asked whether the location of the osteotomy in relation to the knee joint line (3 and 4 cm distally to it) influences the wedge basis length in digital planning. This study demonstrated no statistically significant difference in wedge basis length or in wedge angle. To our best knowledge, this is the first study analysing possible discrepancies in osteotomy gap height or amount of correction when the location of the osteotomy starting point does not exactly match the initial digital planning. The research question of this study is important and clinically relevant because the procedure aims to reach exactly the preoperatively planned alignment correction. Measuring is as good and precise as the devices used. A metal wedge as an osteotomy gap measuring device (Synthes, Solothurn, Switzerland) can be precise within 0.5 mm. With a corpectomy calliper (Synthes, Solothurn, Switzerland), the accuracy is even more precise at 0.1 mm. Accuracy studies comparing the intended to the realized correction are available clinically and in cadaveric studies [10, 15, 16]. Surgical accuracy was about 2°, and could not be improved by navigation [10]. To the best of our knowledge, studies comparing the planned and the realized wedge are not available. The results of the current study demonstrated that the planned osteotomy gap height can be used when the osteotomy is performed within 1 cm above or below the planned osteotomy. The amount of bone loss depending on the thickness of the saw blade used for the main osteotomy cut should of course be considered.

The osteotomy site cannot be chosen without consideration of the bony and surrounding soft tissue anatomy as well as plate dimension and design. Technical notes are available, and provide clear descriptions of how to protect the surrounding anatomical structures [9]. Usually, the OWHTO is performed in a biplanar fashion, and the main osteotomy cut is made proximal to the pes anserinus at the deepest point of the bony concavity of the proximal tibia after partial or complete release of the superficial medial collateral ligament (sMCL). In OWHTO, not only the coronal plane is to be considered, but also the sagittal and axial planes. The position of the hinge significantly affects the posterior tibial slope. Therefore, cadaveric studies emphasize accurate hinge positioning to prevent complications such as hinge fractures or changes in posterior tibial slope or mMPTA [17]. The starting point of the osteotomy also affects the angulation corrections axis (ACA). Following the rules by Paley et al., the ACA should be as close as possible to the centre of rotation and angulation (CORA) to avoid secondary deformities [6, 18].

Because interrater differences were reported to be high in digital planning studies [19], we ruled out interrater inaccuracies by planning all 50 osteotomies by one experienced surgeon. The simple study design is a strength but also a limitation of the study. Only the digital planning accuracy is considered. Since this planning is only two-dimensional, problems arising in a 3D context are not by definition. A 3D approach to this key question with a 3D planning software as well as the surgical implementation and measuring in a cadaver study could be conducted to validate the current results.

## Conclusion

When performing an OWHTO aiming towards the tip of the fibula, the osteotomy starting point does not need to match the planned starting location of the osteotomy exactly. A starting point 1 cm more distal or proximal than what was determined by he digital planning does not alter the size of the osteotomy gap needed to reach the desired amount of correction.

## Data Availability

The datasets used and/or analysed during the current study are available from the Prof. Konrads Christian (christian.konrads@gmail.com) on reasonable request.
